# Effect of impaction energy on dynamic bone strains, fixation strength, and seating of cementless acetabular cups

**DOI:** 10.1002/jor.24418

**Published:** 2019-08-02

**Authors:** Ruben Doyle, Richard J. van Arkel, Jonathan R. T. Jeffers

**Affiliations:** ^1^ Department of Mechanical Engineering Imperial College London London SW7 2AZ United Kingdom

**Keywords:** energy, fixation, fracture, impaction, stability

## Abstract

Seating a cementless acetabular cup via impaction is a balancing act; good cup fixation must be obtained to ensure adequate bone in‐growth and cup apposition, while acetabular fracture must be avoided. Good impaction technique is essential to the success of hip arthroplasty. Yet little guidance exists in the literature to inform surgeons on “how hard” to hit. A drop rig and synthetic bone model were used to vary the energy of impaction strikes in low and high‐density synthetic bone, while key parameters such as dynamic strain (quantifying fracture risk), implant fixation, and polar gap were measured. For high energy impaction (15 J) in low‐density synthetic bone, a peak tensile strain was observed during impaction that was up to 3.4× as large as post‐strike strain, indicating a high fracture risk. Diminishing returns were observed for pushout fixation with increasing energy. Eighty‐five percent of the pushout fixation achieved using a 15 J impaction strike was attained by using a 7.5 J strike energy. Similarly, polar gap was only minimally reduced at high impaction energies. Therefore it is suggested that higher energy strikes increase fracture risk, but do not offer large improvements to fixation or implant‐bone apposition. It may difficult be for surgeons to accurately deliver specific impaction energies, suggesting there is scope for operative tools to manage implant seating. © 2019 The Authors. *Journal of Orthopaedic Research* ^®^ published by Wiley Periodicals, Inc. on behalf of Orthopaedic Research Society. J Orthop Res 37:2367–2375, 2019

Cementless total hip arthroplasty (THA) offers many advantages over cemented THA and is increasingly popular worldwide.^1^, ^2^, ^3^ While cemented prostheses rely upon a polymer cement to hold implants in place, cementless implants rely on press‐fit fixation and high friction generated by an implant with high surface roughness. With experience, cementless procedures deliver excellent long term results.^4^ To implant cementless devices large forces must be generated by the surgeon, typically with a surgical mallet; a process known as impaction. Impaction technique is critical to implant longevity as enough energy must be used in order to seat the implant properly,^5^, ^6^, ^7^, ^8^ but not so much that the bone is fractured or excessively compressed^8^, ^9^, ^10^, ^11^, ^12^, ^13^ or the implant excessively deformed.^14^, ^15^, ^16^ Despite impaction being used in the majority of approximately 100,000 THAs performed per year in the United Kingdom, very little literature exists to guide surgeons to use appropriate energies.

Acetabular fracture is very often not detected during surgery^17^ and while less common than femoral fracture, is more likely to have adverse outcomes.^18^ Fractures of the acetabulum are commonly attributed to the impaction process,^8^, ^10^, ^19^, ^20^ but dynamic bone strains *during* the impaction event are yet to be characterized. In addition, excessive strain in the acetabulum, even when not causing fracture, may cause large deformation of the acetabular cup^14^, ^15^, ^16^ and hinder liner seating,^21^ or cause excessive wear,^14^ or even cup damage.^5^

Biomechanics of acetabular cup fixation has been investigated previously in the literature. As an implant is forced into the bone cavity elastic strain is created by deforming the acetabulum wall through a “pinching” action of the ischial and ilial columns.^22^, ^23^, ^24^, ^25^, ^26^ The hoop stresses generated, together with sufficient quality bone and a rough implant surface provide the initial stability required for bone ingrowth.^27^, ^28^, ^29^ With good cup fixation, micromotion is reduced and bony ingrowth can occur.^30^ Good apposition between bone and implant is also required for the bone to fill any gaps and anchor the implant securely.^31^ Gaps of more than 2 mm have been shown to contribute to early migration,^32^ a possible contributor to aseptic loosening,^33^, ^34^ the most common cause of THA implant revision.^4^

Therefore, to maximize chances of success, a surgeon should seek to maximize fixation and implant seating whilst minimizing the risk of fracture. This study seeks to determine how the impaction energy used by surgeons may affect these parameters. “How hard should I hit?” is a commonly asked question by surgeons, which we can translate into the engineering question: How does impaction energy affect the risk of fracture (quantified by measuring dynamic bone strains), cup deformation (quantified by measuring cup strains), cup fixation (quantified by pushout force and post‐strike strain), and cup seating (quantified by measuring polar gap)? This study answers these questions to build models of seating mechanisms and make surgical recommendations for seating energies.

## METHODS

### Experimental Setup: Drop Rig, Synthetic Bone Model, Acetabular Cups

**Figure 1:**
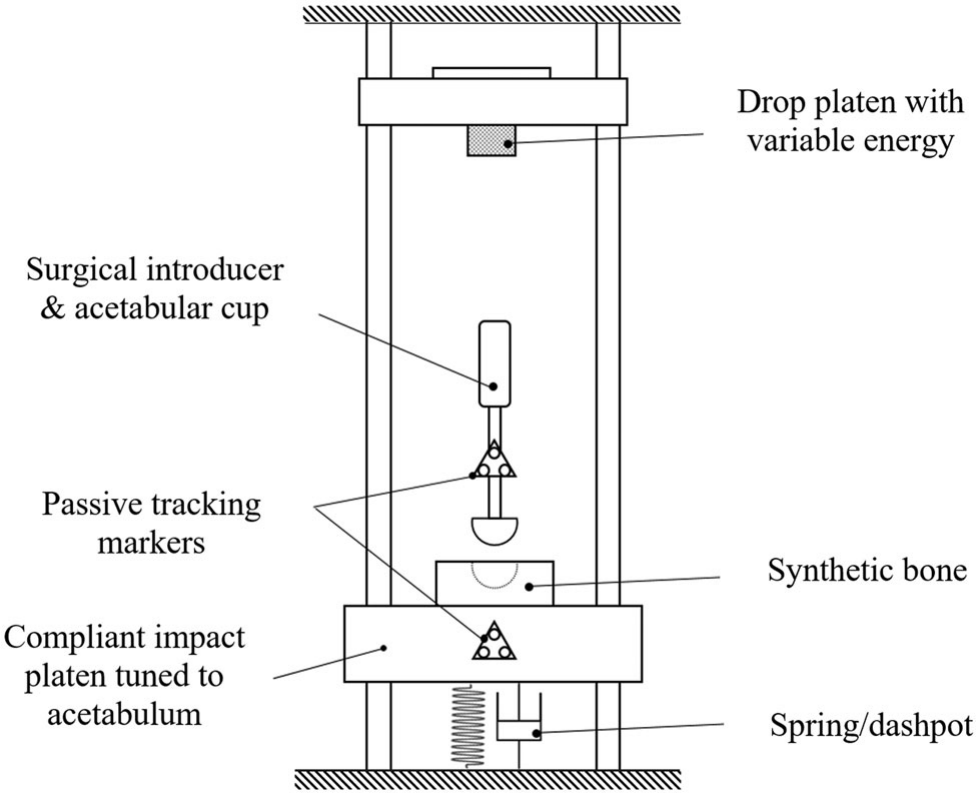
The experimental setup: the drop platen enabled repeatable hammer strikes to be applied with known energy. The spring and dashpot were previously tuned^35^ to replicate the intraoperative movement of the pelvis following impaction.

An in‐vitro drop rig was used to impact the cups into synthetic bone models in a controllable and repeatable manner. A drop weight, representing the surgical mallet, could be adjusted for both height and mass to vary the energy of the impact (Fig. 1). The synthetic bone model was attached to an impaction platen which was supported by a spring/dashpot/mass tuned to provide the same response as the human acetabulum during intraoperative impaction.^36^ A load cell (200C20, PCB Piezotronics, Buffalo, NY) was attached to the lower surface of the drop platen to measure peak impact load during the strike. Load data were acquired at 51.2 kHz using a high‐speed USB data acquisition unit (NI9327; National Instruments, Newbury, UK).

**Figure 2:**
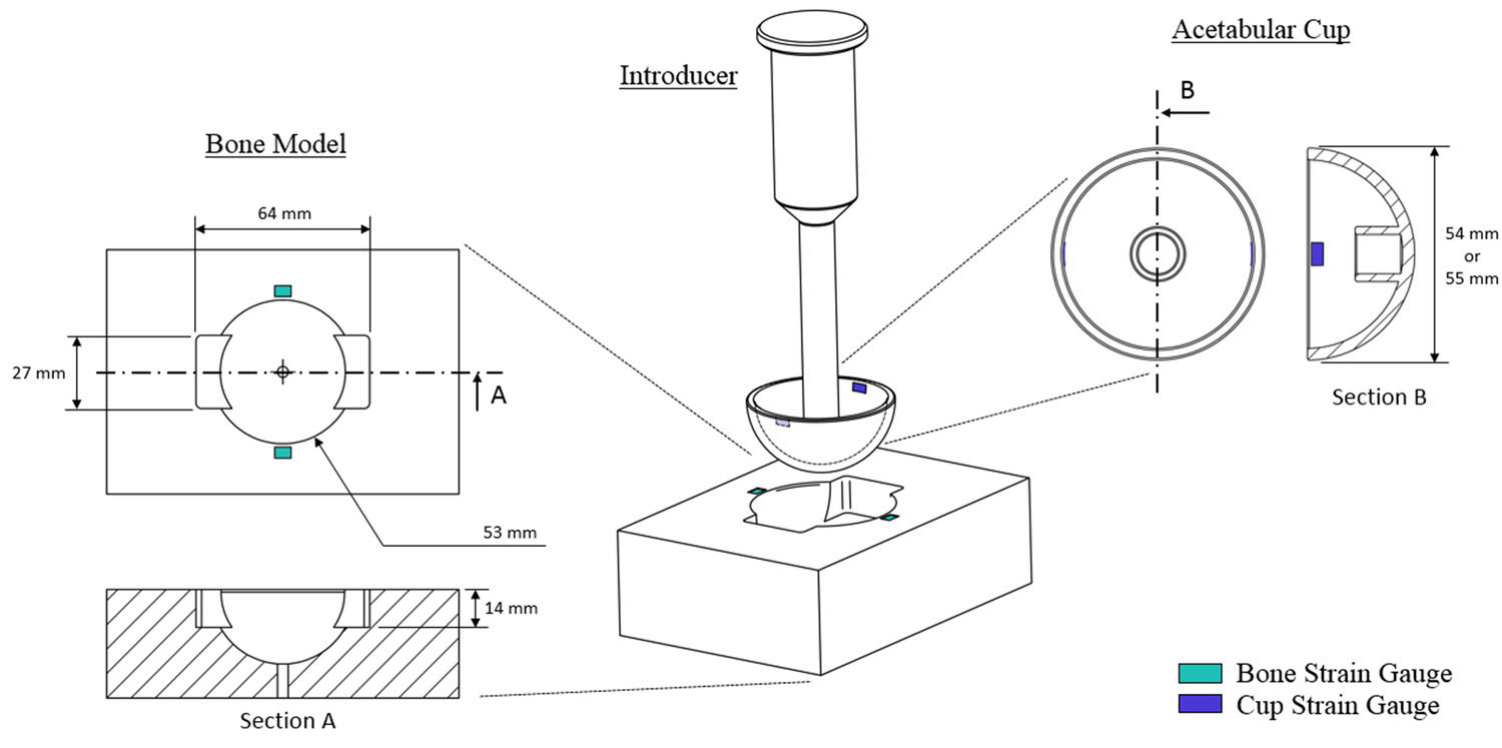
Photograph of the Ti acetabular cup surface finish created in a single part (i.e., no coating) by powder bed fusion additive manufacture on a Renishaw AM250 machine.

The synthetic bone model used was manufactured from 15 PCF (pounds per cubic foot) and 30 PCF Sawbone (#1522‐02 and #1522‐04, Sawbones; Pacific Laboratories, Malmoe, Sweden) to represent low and high bone density. A representative acetabular cavity^25^, ^35^ was CNC milled with a 53 mm ϕ hemispheric cavity centered 1 mm below the top surface of the block (Fig. 2). This simulated cavity has two cutouts to create a highly simplified representation of the acetabular bone, but cup deformation in this model is similar to that measured in cadaver tests.^25^ The resulting hemispheres were measured to be Ø 52.88 mm (SD 0.086).

**Figure 3:**
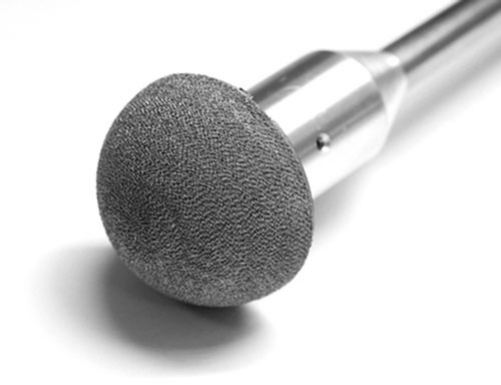
Photograph of the Ti acetabular cup surface finish created in a single part (i.e., no coating) by powder bed fusion additive manufacture on a Renishaw AM250 machine. [Correction added on 08 August 2019; Figure 3 legend was corrected]

The acetabular cups were manufactured from Ti‐6Al‐4V in 54 and 55 mm sizes (Fig. 3). [Correction added on 08 August 2019; Fig. 2 was corrected to Fig. 3]. The 54 mm cups were impacted into the high‐density foam forming a 1 mm interference, while the 55 mm cups were impacted into the low‐density foam producing a 2 mm interference. These were chosen to represent clinical practice where it is common to use a higher interference in low‐density bone. The cups were manufactured by powder bed fusion additive manufacture (AM250, Renishaw, Wotton‐under‐Edge, UK) with a surface roughness created by pseudo‐random sinusoidal outer profiles to represent that of plasma spraying (Rz > 500 μm)^37^ and a rim‐to‐rim stiffness similar to commercially available models.^16^ As such, this model represents around 20,000 (30%) of the cementless acetabular cups implanted in the United Kingdom every year.^1^

### Testing Protocol

Cups (N = 5) were impacted into the low and high‐density synthetic bone using the drop rig, with strain, displacement and impact load data recorded for 10 strikes. The range of impact energies used were defined by the impact energy measured by a senior consultant orthopedic surgeon performing total hip replacement in a cadaver test and hitting as hard as he would in surgery, as gently as he would and somewhere in between. The mallet mass was 0.7 kg (Table 1).^36^ Following implantation with 10 strikes, the displacement data were analyzed to determine when each cup could be considered seated. Each cup was considered seated with the cup progressed by no more than 0.1 mm for the following strike: referred to as the optimum number of impacts. Subsequently, for each of the seven energy levels, cups were impacted into virgin synthetic bones using the optimum number of impacts and pushout tests conducted.

**Table 1:** The Impaction Variables Studied: Both Increases in Drop Mass and Velocity Were Used to Increase the Impaction Energy.

Energy Level	1	2	3	4	5	6	7
Drop mass (kg)	0.6	1.2	1.8	1.2	1.8	1.2	1.8
Drop velocity (m/s)	1.5	1.5	1.5	2.75	2.75	4	4
Energy (J)	0.7	1.4	2.0	4.5	6.8	9.6	14.4

In total 140 tests were performed: 70 to establish the optimum number of strikes for each testing condition, and then 70 to test pushout fixation following impaction with the optimum number of strikes. Virgin synthetic bone samples were used for each of the 140 tests.

### Strain Measurement

Strain was measured using linear strain gauges (350 ohms, SGT‐3F/350TY11; Omega, Manchester, UK), glued to opposing sides of the foam block, 2 mm from the edge of the cavity (Fig. 2). [Correction added on 08 August 2019; Fig. 3 was corrected to Fig. 2]. The location of the strain gauges was based on a pilot test of N = 6 samples, where Digital Image Correlation (DIC) was used to identify the region of peak hoop stress in the bones samples during implant seating. A further two gauges were glued to opposing sides of the internal acetabular cup rim. Gauges were orientated to capture tensile hoop strain in the synthetic bone and compressive hoop strain in the acetabular cup. Cups were positioned so that the cup gauges were at a 90° offset circumferentially to the synthetic bone gauges to measure the main “pinching” effect of the acetabulum (Fig. 2). [Correction added on 08 August 2019; Fig. 3 was corrected to Fig. 2]. Strain data were acquired using the same 51.2 kHz high‐speed USB data acquisition system as the load data (NI9327; National Instruments, Newbury, UK).

### Pushout Fixation

Pushout tests were conducted using a uniaxial load testing machine (model 5565; Instron, High Wycombe, UK). Cups were pushed out using a 6 mm diameter steel pin through a 7 mm entry hole milled into the rear of the synthetic bone. Pushout was conducted at 0.5 mm/min.^38^ Pushout was chosen as a simple measure of fixation that well represents the same trends as a more sophisticated micromotion test.^35^

### Cup Seating

To monitor the position of the acetabular cup in relation to the synthetic bone following each strike, the setup included infrared motion trackers, fixed to the cup introducer and synthetic bone. A motion capture camera (Polaris Vega, NDI, ON, Canada) was used to record the position of both trackers following each strike.

### Data Analysis

Risk of fracture was quantified by correlating the dependent variables of peak bones strain and implantation force with the independent variable of energy per strike. Cup deformation was quantified by correlating the dependent variable of post‐strike cup strain with pushout fixation. The strength of fixation was quantified by correlating the dependent variables of pushout fixation with the independent variable of energy per strike. Cup seating was analyzed by correlating the final polar gap with the independent variable of energy per strike.

For each dependent variable, two types of least‐squares trendline fits were evaluated to characterize the relationship and to identify any limits of the dependent variable (e.g., is there a maximum strength of fixation that can be achieved?). Both were chosen based upon their application to real‐world problems and previous use.^39^ The first was a linear trendline.

**Figure 4:**
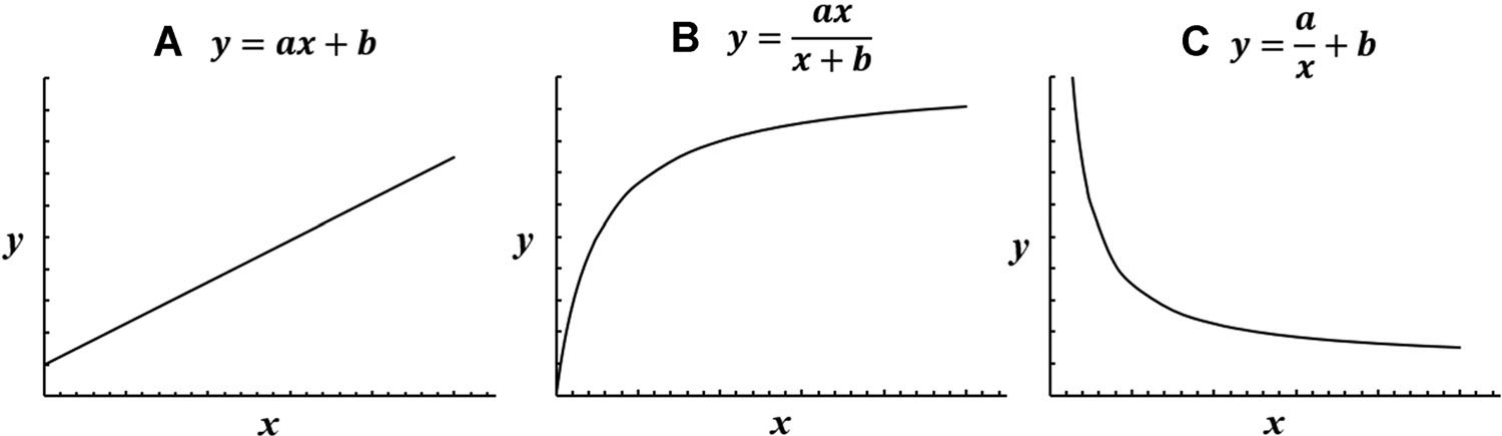
Graphical representation of different fits. (A) Increasing linear (positive α), (B) decreasing linear (negative α), (C) direct asymptotic, (D) inverse asymptotic.

(1) y=αx+β

where *y* is the dependant variable, *x* the independent variable, with *α* and *β* constant*s*. As *x* becomes large, *y* scales directly, either linearly increasing or decreasing depending on whether *α* is found to be positive or negative, respectively (Fig. 4A). A linear fit has no upper/lower limit, and therefore cannot account for phenomena that tend to a maximum or minimum value.

The second trendline fit was a hyperbolic asymptotic test, to determine if the data tended toward a maximum/minimum value and to calculate these limits (Fig. 4A and B).

(2) $$y=\frac{\alpha x}{\beta +x}$$

where *y* is the dependent variable, *x* the independent variable and *α* and *β* are constants determined during the fitting process. For Equation (2), as *x* becomes large, *y* tends towards the maximum *α*.

A least‐squares method was used to fit both Equations (1) and (2) to each relationship using the curve fitting toolbox in MATLAB (2015a; MathWorks, Natick, MA) with a zero *y*‐intercept for all dependent variables apart from the polar gap. To assess the quality of fit for asymptotic fits the data were transformed to be linear, by inverting *x* and *y* data.

### Statistical Analyses

Regression analysis, conducted in MATLAB, was used to assess the quality of data fitting for each relationship reported. Significance level was set to *α* = 0.05. Differences between impact force data for low and high‐density synthetic bone were analyzed using paired *t* tests, conducted in SPSS (v24; IBM UK, Portsmouth, UK).

## RESULTS

**Figure 5:**
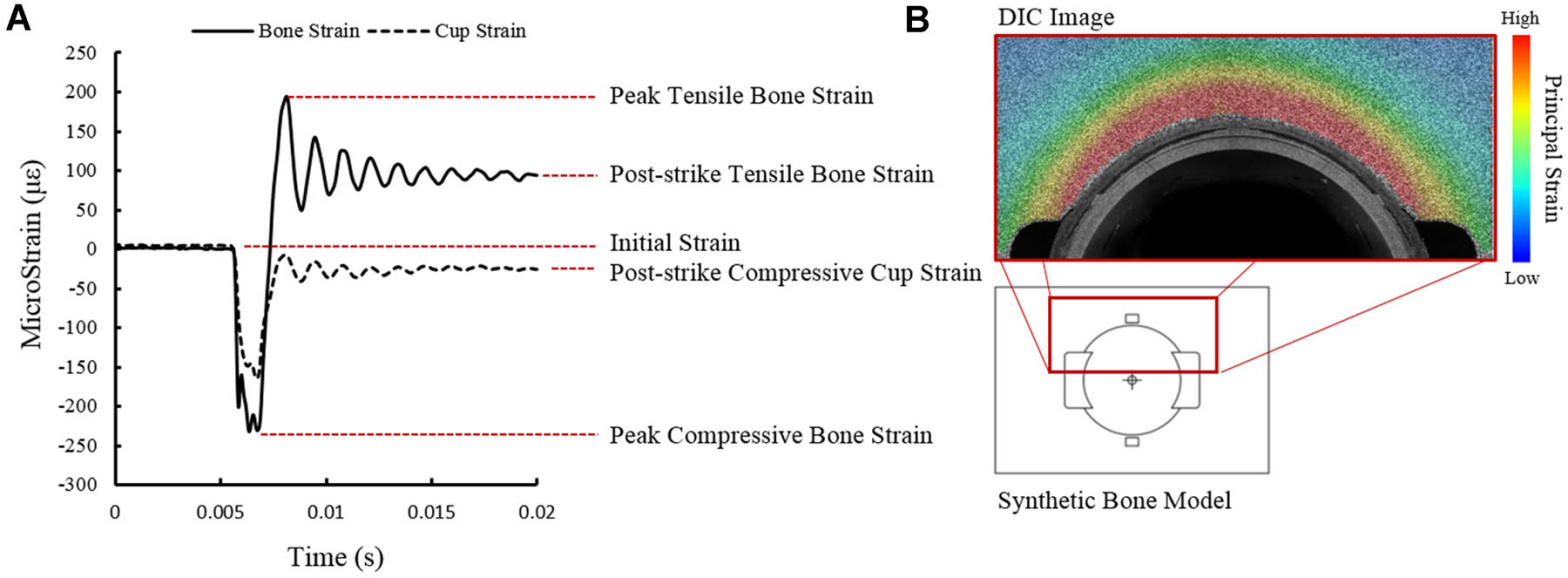
(A) Strain behavior during impaction in cup (solid line) and acetabular cup (dashed line). Peak tensile, peak compressive and post‐strike strain are identified in the bone. Post‐strike strain is recorded in the acetabular cups. (B) DIC image of strain in synthetic bone during seating used to validate strain gage placement. [Color figure can be viewed at wileyonlinelibrary.com].

Two distinct patterns were observed for the behavior of dynamic strain in the bone and acetabular cups (Fig. 5A). In the bone a compressive peak was immediately followed by a tensile peak, followed by a period of oscillation decaying to a final post‐strike tensile strain. Both peak tensile and peak compressive peaks in the synthetic bone are of interest as they are both possible contributors to bone fracture. We consider the compressive peak is caused by the surface deforming in the direction of the impacted cup, while the tensile peak is hoop tensile strain. The strain in the cup exhibited a compressive peak in the hoop direction. Similarly, a period of oscillation preceded the final, post‐strike strain.

Post‐seating, an increase in principal hoop tensile strain was observed nearer to the cup rim (Fig. 4B). Strain was greatest closest to the cup rim and was near axisymmetric.

The peak force scaled linearly with strike energy in both density synthetic bones (*R* ^2^ > 0.97, *p* < 0.005, Table 2). An inverse linear relationship was observed between strike energy and optimum number of strikes to seat in both bone densities (*R* ^2^ = 0.67 and *R* ^2^ = 0.82, *p* < 0.005). The cups only required two strikes to seat at the highest impaction energy, while the full ten strikes were required in the lowest energy group.

**Table 2:** Impact Force and Strikes Required to Seat for Each Energy Level

		Energy Per Strike (J)						
	Bone Density	0.7	1.4	2.0	4.5	6.8	9.6	14.4
Force (kN ± SD)	15	3.9	5.1	7.2	10.8	12.6	14.2	19.5
		(±0.4)	(±0.4)	(±0.6)	(±0.6)	(±1.4)	(±1.9)	(±1.4)
	30	4.2	5.5	7.7	11.7	15.2	15.0	20.6
		(±0.4)	(±0.8)	(±0.2)	(±0.3)	(±1.3)	(±1.2)	(±1.3)
Strikes required to seat	15	10	10	7	3	2	2	2
	30	7	7	8	7	3	3	2

**Figure 6:**
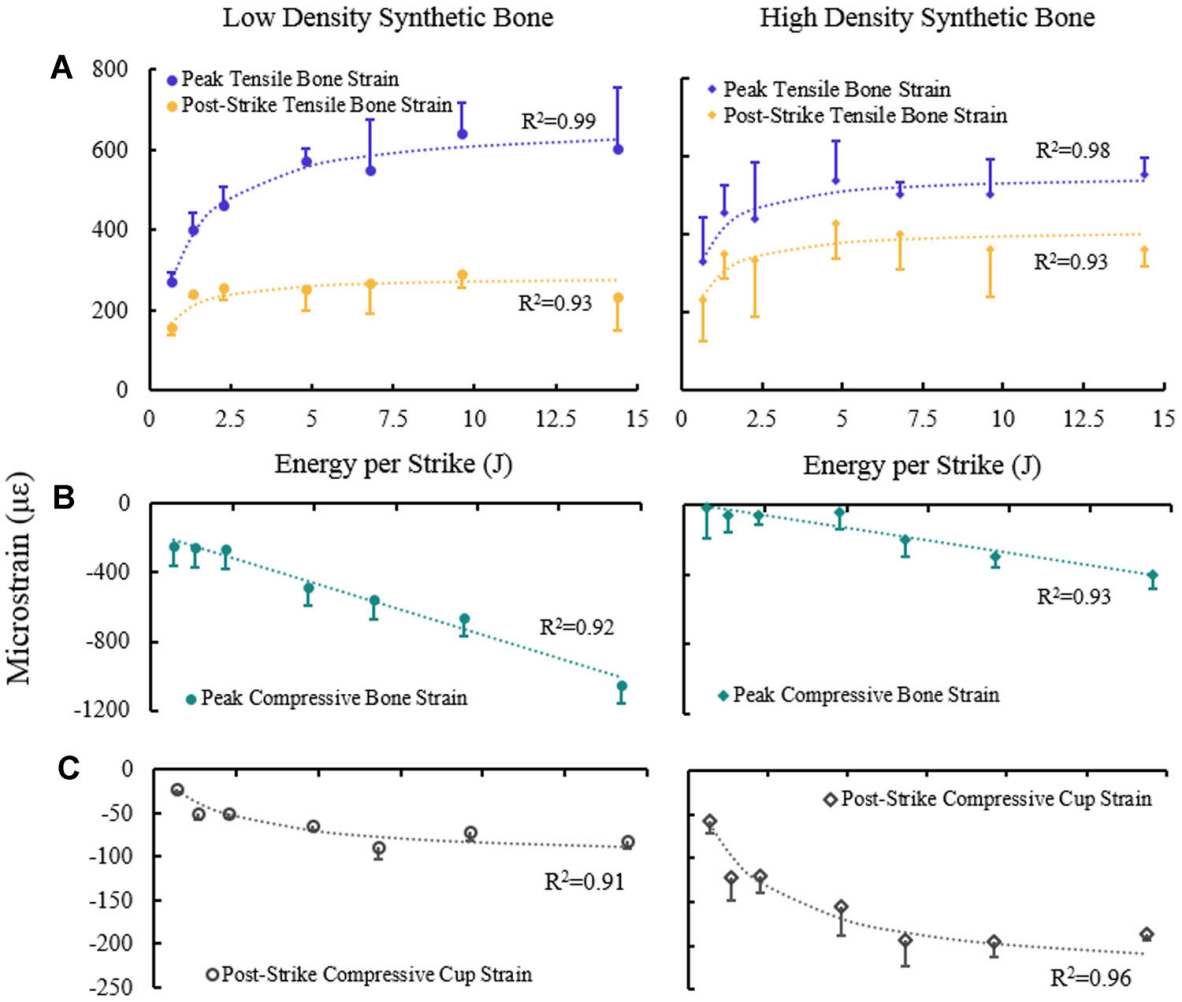
Relationships between energy per strike and (A) peak tensile bone strain, post‐strike tensile bone strain (B) peak compressive bone strain, and (C) post strike compressive cup strain (mean with standard deviations). Peak tensile bone strain, post‐strike tensile bone strain, and post strike compressive cup strain all closely fit an asymptotic trend, suggesting a limit to these strains even at very high energies. Peak compressive bone strain closely fit a linear trend, suggesting very high strain at very high energies. [Color figure can be viewed at wileyonlinelibrary.com].

Relationships between the strain profile (Fig. 4A) and different strike energies were identified (Fig. 6). Peak tensile bone strains, post‐strike bone strains, and post‐strike compressive cup strain were all best described by a direct asymptotic function, tending to a finite limit value as energy tended to large magnitudes (Fig. 6A and C, Table 3). The exception was maximum compressive strain strike energy which was best described by a direct linear relationship (Fig. 6B, Table 3).

**Table 3:** Summary of Results of Tested Fits for Each Relationship^*^

		Variable		Linear		Aysmptotic	
Bone Density	Figure	Independent	Dependant	*R* ^2^	Limit	*R* ^2^	Limit
Low	4A	Energy per strike	Peak tensile bone strain	0.65	∞	0.99	662
	4A	Energy per strike	Post‐strike tensile bone strain	0.55	∞	0.93	277
	4B	Energy per strike	Peak compressive bone strain	0.92	∞	0.90	2,000
	4C	Energy per strike	Post‐strike compressive cup strain	0.68	∞	0.91	91.7
	6	Energy per strike	Pushout fixation	0.81	∞	0.93	692
	7A	Post‐strike compressive cup strain^a^	Pushout fixation	0.95	∞	0.94	>10^5^
	7B	Post‐strike tensile bone strain^a^	Pushout fixation	0.74	∞	0.66	>10^5^
	8B	Energy per strike	Polar gap	0.34	∞	0.81	1.10
High	4A	Energy per strike	Peak tensile bone strain	0.59	∞	0.98	550
High	4A	Energy per strike	Post‐strike tensile bone strain	0.55	∞	0.93	405
	4B	Energy per strike	Peak compressive bone strain	0.93	∞	0.91	>10^5^
	4C	Energy per strike	Post‐strike compressive cup strain	0.69	∞	0.96	217
	6	Energy per strike	Pushout fixation	0.78	∞	0.96	1,440
	7A	Post‐strike compressive cup strain^a^	Pushout fixation	0.97	∞	0.96	>10^5^
	7B	Post‐strike Tensile bone strain^a^	Pushout fixation	0.78	∞	0.73	>10^5^
	8B	Energy per strike	Polar gap	0.28	∞	0.92	0.45

^*^Fit coefficients, *R* ^2^ values, *p*‐values and limits when *x* tends to infinity are given in Table 3. Peak and post‐strike tensile strain, post‐strike compressive strain, polar gap, and the pushout fixation were all best described by an asymptotic relationship (higher *R* ^2^), indicating that there was an upper limit to these variables during impaction. However, peak compressive bone strain was better described by a linear fit: compressive strain fracture risk increased linearly with impaction energy. Chosen fits are highlighted.

^a^ Dependant, not independent variable. *R* not *R* ^2^ reported.

Peak tensile bone strain was on average 2.1× larger than post‐strike tensile bone strain in the low‐density synthetic bone and 1.4× larger in the high. In the most extreme case, a 15 J strike in low‐density bone produced a peak tensile bone strain 3.4× larger than post‐strike tensile bone strain. The bone therefore experienced much higher strain during the impaction event than the steady‐state conditions post‐strike.

**Figure 7:**
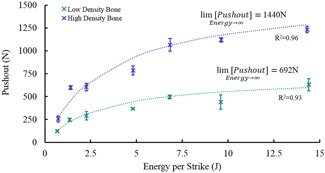
Pushout fixation for cups impacted with different energy levels (mean with standard deviations). An asymptotic relationship is fitted implying a limit to the amount of fixation that can be achieved even with extremely high impaction energy. [Color figure can be viewed at wileyonlinelibrary.com].

The relationship between energy (per seating strike) and pushout fixation (measured after the optimum number of impacts) was best described by a direct asymptotic relationship in both low and high‐density synthetic bone (*R* ^2^ = 0.93 and *R* ^2^ = 0.96, respectively, Table 3, Fig. 7). These relationships imply that even at higher strike energies there is a limit to how much pushout fixation that can be achieved. These limits were found to be 692 and 1440 N for the low and high density bone models respectively.

**Figure 8:**
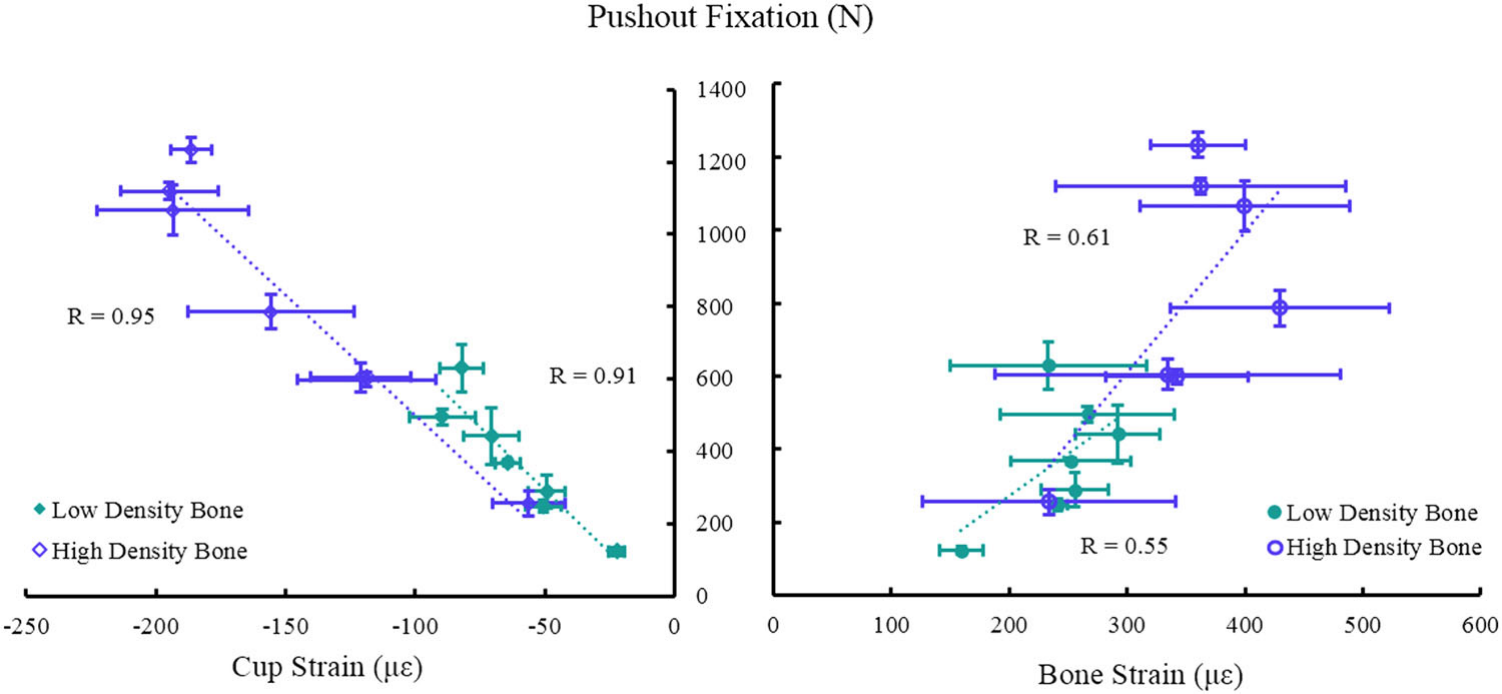
Relationship between pushout fixation and (A) post‐strike compressive cup strain and (B) post‐strike tensile bone strain (mean with standard deviations). Here cup strain is a better indicator of cup fixation than bone strain. [Color figure can be viewed at wileyonlinelibrary.com].

A strong correlation was observed between post‐strike compressive cup strain and pushout force in both bone densities (*R* > 0.91). A weaker correlation was observed between the post‐strike tensile bone strains and pushout force for both bone densities (*R* > 0.55) (Fig. 8).

**Figure 9:**
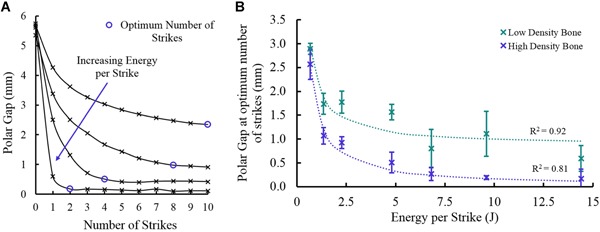
(A) Polar gap at each of the 10 strikes, given for an illustrative range of energies. As energy per strike increases the number of strikes taken to seat is reduced. (B) Final polar gap for each cup when impacted at different energies (mean with standard deviations). The fitted asymptotic relationship suggests that there is a limit to the minimum final polar gap that can be achieved. [Color figure can be viewed at wileyonlinelibrary.com].

A common trend was observed for the polar gap during seating at different energies. For each different energy, implant progression (movement of the cup into the cavity and reduction of polar gap for each strike) was greatest during early strikes, and reduced strike by strike (Fig. 9A). The higher energy strikes reached a seated condition after fewer strikes. (*p* < 0.023, Table 2). The relationship between final polar gap and energy was best described by an inverse asymptotic relationship (Fig. 9B), indicating the polar gap reduces to a limit value and increasing the strike energy further may not progress the cup any further. The minimum polar gap achieved in the low and high‐density bone modelswere 0.60 and 0.17 mm, respectively.

## DISCUSSION

This study demonstrated for the first time that peak strain in the bone during the impaction event can be several times higher than the post‐impaction steady‐state strain. Our 2 mm diametrical interference in low‐density bone is in line with many manufacturers’ recommended surgical techniques but generates a higher peak bone strain during impaction than a 1 mm press‐fit in higher density bone. Higher peak strains in a bone increase risk of bone fracture and should be considered by manufacturers and surgeons when deciding impaction technique. A second finding from this study was the law of diminishing returns applies to the amount of fixation strength and implant seating that can be achieved by increasing impaction energy. For example, at just 7.5 J, 85% and 84% of the total pushout available at 15 J was achieved (for low and high‐density synthetic bone, respectively). Similarly, the same doubling of impaction energy only reduced the polar gap by around 0.1 mm. The necessary gaps of <2 mm (which have been shown to be tolerable clinically^16^, ^32^) were achieved in both bone densities at 7.5 J. These data underpin the development of impact‐limiting instrumentation which, together with improvement in defining the amount of press‐fit required for each individual patient, will improve hip replacement surgical technique.

The limit to the amount of implant fixation that can be achieved for a fixed bone stiffness and interference fit could be explained by the phenomenon of bone damage and compression that occurs during implantation that has previously been observed.^40^ It has been shown that plastic deformation occurs locally around the cup surface. The higher the impact forces used, the more deformation that may occur and therefore less elastic energy is stored. The asymptotic behavior of tensile bone strains does imply that hoop tensile bone stresses are self‐limiting. However compressive peak strains appeared to scale linearly with energy, without a predicted upper limit. This may be an underlying mechanism for acetabular fracture during impaction.^9^, ^18^

Another important finding of this study was the identification of a strong linear relationship between pushout fixation and the deformation of the cup itself. During seating of an implant, it would be beneficial to maximize fixation as much as possible. However, it would also be beneficial to minimize compressive cup strains in order to reduce component deformation (to lessen the chances of excessive component wear, or improper liner seating). However, this study shows that these factors are inextricably linked—to generate fixation you must generate strain. The force of the “pinch” of bone on cup relates to the amount of fixation and stability the cup receives.^15^, ^16^, ^41^, ^42^ Cup deformation might be reduced by increasing implant wall stiffness. However, it is well documented that excessive implant stiffness leads to strain shielding, an undesirable function of bone.^43^ This appears to be a trade‐off: a balance between component deformation and stress shielding must be determined.

Use of synthetic bone will have influenced our results as the representative geometry and material properties only provide an approximation of the complex anatomy of the pelvis and the intraoperative environment. However, it allowed for experimentation that would not be ethical in patients. A cadaveric tissue model would be more representative of the in‐vivo scenario but the variation between specimens is too great. Variables such as acetabular cup size and positioning, bony morphology and properties, body mass index and hammer strike direction may interact with each other and thus necessitate prohibitively large sample sizes to detect differences in impaction technique. A repeated measures analysis would help mitigate this variation but is not possible for the present study as the bone is damaged by seating a cup, so the same cadaver could not have been used for multiple energies. Our impaction rig with a synthetic bone model allowed us to control all these variables and thus isolate the effects of impaction energy. The rig has been previously been validated through direct comparison to cadaveric THA in eight hips; an important aspect of this validation was replicating the intraoperative boundary conditions to replicate the physiological movement of the acetabulum away from the surgeon following a hammer strike.^36^ The energy of the strikes tested also represent the region measured operatively (1–15 J), as previously reported.^5^, ^36^ Sawbones have been shown to model the mechanical properties of biological tissues^44^, ^45^, ^46^ and result in cup fixation stability within the range measured in cadavers.^47^ Previous literature demonstrates the value of these models to draw important conclusions.^23^, ^37^, ^48^ To account for variation in bone properties, we modeled two bone densities based upon previous literature.^6^, ^35^, ^45^, ^49^, ^50^ We used a different press‐fit for each bone density which limits the comparisons between our different density models, but these were chosen to represent the lower press fit that would be used clinically for the more dense bone. The milled bone cavity has also been previously demonstrated to be a surrogate for the anatomy of the acetabular socket.^25^

Many brands of the cementless shell are used clinically,^1^ and thus we elected to use a representative acetabular cup rather than tailor the results to a specific device manufacturer. It is likely that different devices/coatings will alter the numeric values for the limits for minimum polar gap and pushout fixation strength. However, these changes are unlikely to influence the main findings as these are a result of the mechanics of press‐fit impaction and the properties of bone. An advantage of our method is it provides a controlled model for measuring differences between devices and could provide a useful means to compare a new device/coating against a clinical gold‐standard.

In orthopedic surgery, press‐fit implant fixation is a proven technique, but achieving primary fixation is critical for long term success. The intraoperative impaction that creates the primary fixation is highly uncontrolled compared to the manufactured precision of the implant and bone preparing instruments. This paper has highlighted important features of the strain that occurs in bone during the impaction event and demonstrated the diminishing returns of increasing energy to achieve fixation and seating. These data could lead to substantial improvement in the impaction process, allowing surgeons to reliably seat implants with minimum energy, and thus avoid/minimize adverse loading on the bone. Clinically, caution should be exercised with high energy impaction: moderate energy strikes may prove just as beneficial and reduce the chance of fracture.

## AUTHORS’ CONTRIBUTION

All authors have contributed significantly to the elements of this study. Study design was conducted by RD. and JJ. Experiment setup was conducted by RD. and RVA. Data analysis was conducted by RD. Manuscript writing and revision was conducted by RD., RVA., and JJ. All authors have read and approved the final manuscript.
